# Histologic Heterogeneity of Metastases in Clear Cell Renal Cell Carcinoma with Sarcomatoid Differentiation

**DOI:** 10.3390/jcm15103959

**Published:** 2026-05-21

**Authors:** Kaitlin Berry, William Paul Skelton, Madison Karabinus, Steven Monda, Raina Tandon, Henry Frierson, Allison M. May

**Affiliations:** 1 Department of Urology, University of Virginia, Charlottesville, VA 22903, USA; 2 Department of Medicine, University of Virginia, Charlottesville, VA 22904, USA; 3 Department of Pathology, University of Virginia, Charlottesville, VA 22903, USA; 4 Department of Urology, University of Michigan, Ann Arbor, MI 48109, USA

**Keywords:** renal cell carcinoma, kidney cancer, sarcomatoid renal cell carcinoma, metastasis, rhabdoid renal cell carcinoma, clear cell renal cell carcinoma

## Abstract

**Background/Objectives**: Sarcomatoid or rhabdoid renal cell carcinoma (sRCC) represents an aggressive dedifferentiated phenotype of RCC associated with high metastatic potential. The histologic composition of metastatic lesions arising from clear cell RCC with sarcomatoid/rhabdoid differentiation (ccRCC/sRCC) and its relationship to the primary tumor remain incompletely characterized. **Methods**: We retrospectively reviewed patients undergoing nephrectomy for ccRCC/sRCC who had at least one resected metastatic lesion between 2013 and 2025 at a single institution. Primary and metastatic lesions were characterized by the percentage of clear cell versus sarcomatoid/rhabdoid histology. Associations between sarcomatoid/rhabdoid percentage in the primary tumor, metastatic histology, metastatic location, and overall survival were examined. **Results**: Twenty-six patients with 63 metastases were included. Metastatic histology demonstrated substantial heterogeneity, with 27 lesions (43%) showing pure clear cell histology, 21 (33%) mixed, and 15 (24%) pure sarcomatoid/rhabdoid. Some patients had multiple metastases with differing histology. Increasing sarcomatoid/rhabdoid percentage in the primary was associated with a higher likelihood of sarcomatoid/rhabdoid in metastases (*p* < 0.001). ROC analysis demonstrated primary tumor sarcomatoid/rhabdoid percentage predicted sarcomatoid/rhabdoid differentiation in metastases (AUC 0.84, 95% CI 0.73–0.95). An optimal cutoff of 10% sarcomatoid/rhabdoid differentiation predicted sarcomatoid/rhabdoid features in metastases. Metastatic histology varied by site, with lymph node metastases more frequently demonstrating clear cell morphology and visceral metastases more commonly exhibiting sarcomatoid/rhabdoid features. **Conclusions**: Metastases arising from ccRCC with sarcomatoid/rhabdoid differentiation exhibit marked histologic heterogeneity. These findings highlight the complex biology of ccRCC/sRCC metastasis and underscore the need for studies examining molecular drivers of metastatic heterogeneity, as well as the relationship between metastatic histology and therapeutic response.

## 1. Introduction

Sarcomatoid renal cell carcinoma (sRCC) is an aggressive dedifferentiated form of kidney cancer that can arise across histologic subtypes and is observed in up to 20% of advanced renal cell carcinomas [1]. Histologically, sRCC is characterized by areas of spindle-shaped mesenchymal cells arising within a conventional epithelial RCC, most commonly clear cell RCC (ccRCC). Tumors frequently also exhibit intermixed rhabdoid differentiation, which demonstrates similar aggressive clinical behavior and shares overlapping genomic and transcriptional features with sarcomatoid differentiation [2]. The proportion of sarcomatoid or rhabdoid differentiation within a tumor can vary widely, ranging from focal involvement to near complete replacement of the epithelial component, reflecting significant intratumoral heterogeneity [1].

The presence of sarcomatoid/rhabdoid differentiation is of major clinical importance, as it is associated with high-grade disease, rapid progression, and poor oncologic outcomes, with metastasis rates approaching 80% [3]. At the molecular level, sarcomatoid tumors are characterized by enrichment of alterations in tumor suppressor pathways and upregulation of immune-related gene expression signatures, which may contribute to their aggressive phenotype and distinct therapeutic vulnerabilities. In recent years, this biology has translated into evolving treatment strategies, with immune checkpoint inhibitor-based therapies emerging as the preferred approach for patients with metastatic sRCC. This is exemplified by studies such as the CheckMate 214 trial, which demonstrated improved outcomes with combination immunotherapy in patients with sarcomatoid features [4]. As such, accurate characterization of sarcomatoid differentiation in both primary and metastatic tumors has direct implications for prognosis, treatment selection, and clinical trial stratification.

Despite these advances, important gaps remain in our understanding of the metastatic behavior of sRCC. Common sites of metastasis include lymph nodes, lung, liver, and bone [1], yet the histologic composition of metastatic lesions and the extent to which they reflect the primary tumor remain incompletely defined. Sarcomatoid and rhabdoid differentiation are thought to arise through an epithelial to mesenchymal transition (EMT) [5], a process associated with increased invasiveness, metastatic potential, and cellular plasticity. This raises the possibility that tumor cells may undergo dynamic phenotypic changes during dissemination and colonization of distant sites, potentially leading to heterogeneity in metastatic histology.

Prior studies suggest that metastases arising from tumors with sarcomatoid/rhabdoid differentiation can exhibit a wide range of histologic patterns, including pure epithelial carcinoma, pure sarcomatoid/rhabdoid morphology, or mixed features [6]. However, available data remain limited and are largely derived from small, heterogeneous cohorts that combine multiple primary histologic subtypes, including clear cell, chromophobe, papillary, and others, despite the well-recognized biological and clinical differences among these subtypes [6]. Furthermore, few studies have systematically evaluated multiple metastatic sites within the same patient or examined the relationship between the extent of sarcomatoid/rhabdoid differentiation in the primary tumor and the histologic composition of metastatic disease. As a result, the relationship between sarcomatoid/rhabdoid differentiation in primary ccRCC and the histologic characteristics of metastatic lesions remains incompletely characterized. A better understanding of this relationship may provide insight into tumor evolution, metastatic dissemination, and drivers of therapeutic response and resistance.

In this study, we evaluated a cohort of patients with nephrectomy specimens containing ccRCC with sarcomatoid or rhabdoid differentiation who also underwent surgical resection of metastatic disease. We aimed to characterize the histologic composition of metastatic lesions, assess concordance between primary and metastatic histology, and explore the relationships between the extent of sarcomatoid/rhabdoid differentiation in the primary tumor and metastatic patterns.

## 2. Methods

This retrospective cohort study was conducted at the University of Virginia (UVA) Comprehensive Cancer Center. The study was approved by the UVA Institutional Review Board with waiver of informed consent due to its retrospective design and minimal risk to participants. Patients were identified through institutional pathology and surgical databases. We included patients who underwent nephrectomy between 2013 and 2025 with pathology demonstrating ccRCC containing sarcomatoid and/or rhabdoid features and who had at least one site of biopsied or resected metastasis with slides available for centralized review. Baseline clinical characteristics collected included age at diagnosis, sex, race, body mass index (BMI), and TNM stage. Pathologic features of the primary tumor and all available metastatic lesions were recorded, including the proportion of sarcomatoid or rhabdoid differentiation and histologic subtype.

All hematoxylin and eosin-stained slides from primary and metastatic tumor slides were reviewed independently by two pathologists with genitourinary expertise, both blinded to clinical outcomes, to confirm the presence of clear cell and sarcomatoid histology. Tumors containing rhabdoid features were included in the sarcomatoid category for analysis due to their overlapping biological behavior [2]. The percentage of sarcomatoid/rhabdoid differentiation in the primary tumor was recorded based on the original pathology report. Tumors described as having “focal” or “<5%” sarcomatoid differentiation were assigned a value of 2% for quantitative analyses. Metastatic lesions were categorized based on pathologic review and histologic composition as “pure clear cell”, “predominantly clear cell (>50% clear cell)”, “mixed (approximately equal clear cell and sarcomatoid/rhabdoid)”, “predominantly sarcomatoid (>50% sarcomatoid/rhabdoid)”, and “pure sarcomatoid/rhabdoid”. For statistical analyses, metastatic lesions were further grouped as predominantly clear cell, mixed, or predominantly sarcomatoid/rhabdoid. Primary tumors were categorized as having focal sarcomatoid/rhabdoid pattern (<5%), mixed sarcomatoid (5–80%), or diffuse sarcomatoid/rhabdoid (>80%).

Treatment details including first-line and subsequent systemic therapies were collected when available from the electronic medical record. Clinical outcomes, including disease progression and overall survival, were recorded through the study cutoff date of June 2025. Progression was defined based on radiographic or clinical documentation in the medical record.

Statistical analyses were performed using GraphPad Prism(Version 11.0.1). Continuous variables were summarized using medians and interquartile ranges (IQR) and compared using the Mann–Whitney U test due to non-normal distributions. Categorical variables were compared using Fisher’s exact test or the Fisher-Freeman-Halton exact test for contingency tables larger than 2 × 2. Concordance between primary tumor histology and metastatic histology was evaluated descriptively and using nonparametric comparisons. Receiver operating characteristic (ROC) curve analysis was performed to assess the ability of the percentage of sarcomatoid/rhabdoid differentiation in the primary tumor to predict the presence of sarcomatoid/rhabdoid morphology in metastatic lesions. The optimal cutoff was determined using the Youden index. A two-sided *p*-value < 0.05 was considered statistically significant.

Due to the fact that some patients contributed more than one metastatic lesion, analyses were performed both at the metastasis level (treating each metastasis as an independent observation) and at the patient level (summarizing histology across metastases for each patient). This dual approach was used to account for potential intra-patient correlation and to ensure that observed associations were not driven solely by patients with multiple sample lesions.

Overall survival (OS) was estimated using the Kaplan–Meier method and compared descriptively across histologic groups. Given the limited sample size and heterogeneity in treatment exposure, formal multivariable survival analyses were not performed.

## 3. Results

A total of 30 patients were identified who had undergone nephrectomy with resulting pathologic review showing clear cell renal cell carcinoma with sarcomatoid or rhabdoid features and who also had biopsied or resected metastatic disease from 2013 to 2025 at UVA. Upon pathologic review, 4 patients were excluded due to scant/necrotic tissue precluding definitive histological diagnosis, due to the biopsied specimens. Our final analysis included 26 patients, all of whom had fully surgically resected metastatic tissue with definitive histologic diagnosis. The 26 patients had a collective 63 sites of resected metastases.

The majority of patients (57.7%) were male, and the average age was 62 years (Table 1). The most common sites of metastasis were the regional lymph node (29 sites from 11 different patients), adrenal gland (8 sites from 8 patients), liver (7 sites from 4 patients), lung (4 sites from 4 patients), bone (3 sites from 3 patients), and non-regional lymph nodes (4 sites from 4 patients) (Table 1). The remaining metastatic sites included omentum (1 site), brain (1 site), muscle (2 sites from 2 patients), colon or small bowel (2 sites from 1 patient), pituitary (1 site), and scalp (1 site). Fifteen patients (58%) had two or more sites of resected metastatic disease, and eight patients (31%) had three or more sites. (Table 1). The majority of metastases were synchronous and resected at the time of nephrectomy, while nine (14%) were metachronous and resected subsequently after nephrectomy.

**Figure 1:**
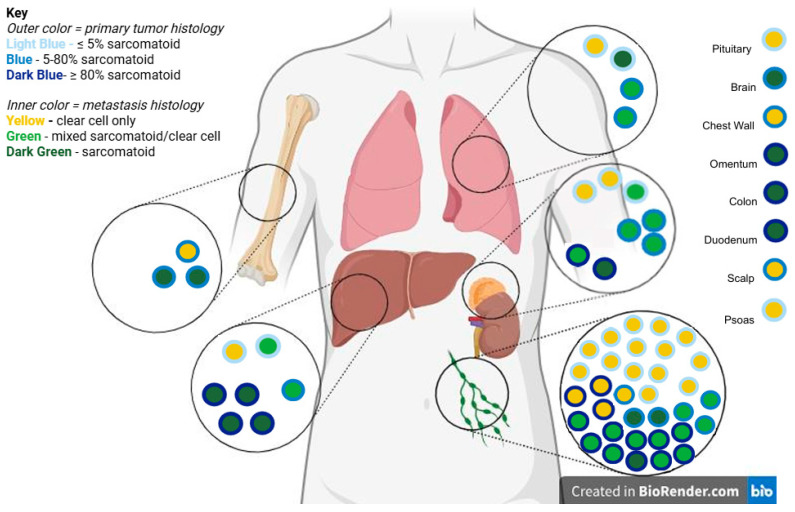
Distribution of primary tumor and metastatic histology across sites. Each circle represents an individual metastatic lesion. The outer ring denotes the percentage of sarcomatoid/rhabdoid differentiation in the primary tumor (<5%, 5–80%, or >80%), while the inner circle represents the histology of the corresponding metastatic site (clear cell, mixed, or sarcomatoid). Metastatic sites with multiple samples (lung, adrenal, liver, lymph node, and bone) are depicted anatomically to illustrate site-specific patterns. Sites represented by a single sample are listed separately on the right. Image created in BioRender. May, A. (2026). https://biorender.com/8o71iew.

Regarding the primary tumor, six patients (23%) were found to have primary tumors harboring diffusely sarcomatoid/rhabdoid features (i.e., greater than 80% sarcomatoid/rhabdoid features), with eleven (42%) having mixed sarcomatoid/rhabdoid features (i.e., between 5% and 80% sarcomatoid/rhabdoid features), and nine (35%) having focal sarcomatoid/rhabdoid features (i.e., <5% sarcomatoid/rhabdoid features; Supplemental Table S1). In regard to metastatic histology, twenty-seven (43%) metastatic lesions showed pure clear cell histology, fourteen lesions (23%) had pure sarcomatoid/rhabdoid histology, and twenty-one lesions (34%) had mixed clear cell and sarcomatoid/rhabdoid histology (Supplemental Table S1). Metastatic locations and histology are depicted in Figure 1.

**Figure 2:**
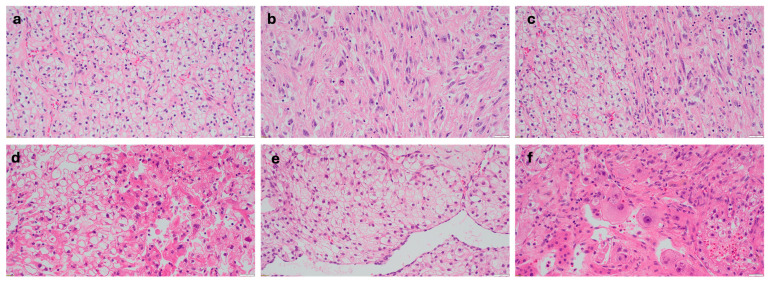
Hematoxylin and eosin-stained slides from primary and metastatic tumors showing examples of heterogeneous histology seen. From patient #10 (**a**) a clear cell region from the primary tumor, (**b**) a sarcomatoid/rhabdoid region from the primary tumor, and (**c**) the adrenal metastasis showing mixed clear cell (left) and sarcomatoid/rhabdoid (right). From patient #5 (**d**), clear cell (left) and sarcomatoid/rhabdoid (right) region from the primary tumor, (**e**) a liver metastasis showing pure clear cell histology, and (**f**) an adrenal metastasis showing mixed clear cell/rhabdoid histology, demonstrating differing histology in metastatic sites from the same patient.

Of the 15 patients with multiple metastatic sites, 13 had the same histologic composition of all metastatic sites. Two patients had metastases with differing histology, including patient #17 who had a brain lesion that was purely sarcomatoid and a separate scalp lesion that was purely clear cell, and patient #5 who had a liver lesion with purely clear cell histology and an adrenal lesion and a second liver lesion, which had a mix of clear cell and rhabdoid (Figure 2).

**Figure 3:**
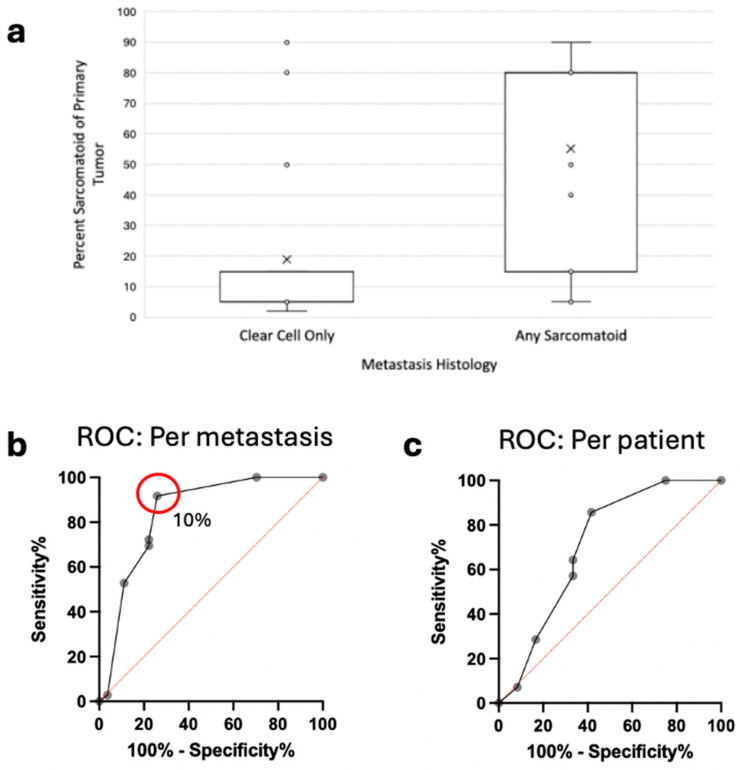
(**a**) Comparison of percentage of sarcomatoid/rhabdoid features in primary tumor associated with metastases containing pure clear cell versus any degree of sarcomatoid/rhabdoid histology (*p* < 0.001), the X denotes the mean. (**b**) ROC analysis of primary tumor sarcomatoid/rhabdoid percentage predicting sarcomatoid/rhabdoid features in associated metastasis, with each metastasis as an independent datapoint (AUC = 0.84, 95% CI 0.73–0.95, *p* < 0.001; the red circle shows the optimal cutoff of 10%). (**c**) ROC analysis of primary tumor sarcomatoid/rhabdoid percentage predicting sarcomatoid/rhabdoid features in associated metastasis, with each patient as a datapoint (AUC = 0.71, 95% CI 0.50–0.92, *p* = 0.068).

There was no significant difference in the degree of sarcomatoid/rhabdoid features in the primary tumors versus metastatic lesions. Among patients with pure clear cell histology in metastases, the median sarcomatoid/rhabdoid percentage in primary tumors was 5% (IQR 5–10) *(n* = 27), compared to 80% (IQR 15–80) among those with any sarcomatoid/rhabdoid features in metastases (*n* = 36) (Figure 3a). This difference was statistically significant (*p* < 0.001, one-sided Mann–Whitney U). We performed a receiver operating characteristic (ROC) analysis at a metastasis level, treating each metastatic lesion as an independent data point, and at the patient level, in which each patient was considered a separate data point to decrease over-representation of patients with multiple metastases. This demonstrated that the percentage of sarcomatoid/rhabdoid differentiation in the primary tumor significantly predicted the presence of sarcomatoid/rhabdoid in metastatic lesions with an area under the curve of 0.84 (95% CI 0.73–0.95, *p* < 0.001). At the patient level, the percent sarcomatoid/rhabdoid in the primary tumor demonstrated moderate ability to predict the presence of sarcomatoid/rhabdoid in the metastases (AUC = 0.71, 95% CI 0.50–0.92, *p* = 0.068, Figure 3).

Prior literature determined a cut-off of 30% sarcomatoid/rhabdoid differentiation in the primary tumor to be predictive of any degree of sarcomatoid in metastatic lesions in a heterogeneous cohort, including clear cell, chromophobe, papillary, and unclassified tumors [5]. Therefore, we explored the 30% cutoff in our cohort, which was comprised clear cell/sarcomatoid tumors. Of 13 patients with <30% sarcomatoid or rhabdoid in their primary tumor, the majority (8/13, 62%) had pure clear cell histology in metastases, although 5 patients had mixed histology with some degree of sarcomatoid/rhabdoid and 1 patient had pure sarcomatoid metastasis. Of 13 patients with >30% sarcomatoid/rhabdoid in their primary, 9 patients (69%) had some degree of sarcomatoid/rhabdoid in their metastases, with 8 of those having predominantly or purely sarcomatoid/rhabdoid histology and 4 having pure clear cell metastases. Based on our ROC analysis, we determined that an optimal cutoff in our ccRCC/sRCC dataset was 10% sarcomatoid in the primary tumor to predict sarcomatoid or rhabdoid in the metastasis (Figure 3b).

Several patients demonstrated discordant metastatic histology that strongly deviated from the above patterns, including one patient with 90% sarcomatoid in the primary who had a metastatic non-regional lymph node with pure clear cell histology, and a patient with 80% sarcomatoid in the primary with two regional lymph nodes with pure clear cell histology. Conversely, a patient with 5% sarcomatoid in the primary had a lung lesion with pure sarcomatoid histology, demonstrating that, although there was a trend towards more sarcomatoid in the primary leading to a higher likelihood of sarcomatoid features in metastases, this association was not necessarily seen in all tumors.

**Figure 4:**
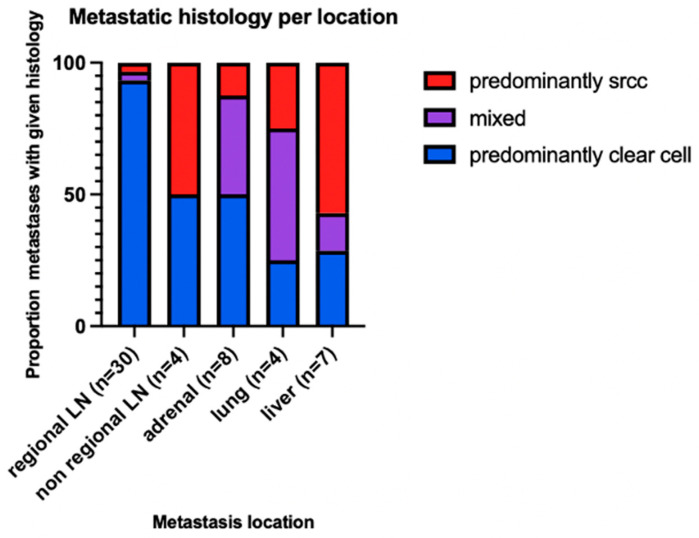
Proportion of metastatic histology characterization based on metastatic location.

To explore whether histology predicted metastatic tropism, we evaluated the distribution of metastatic histology by metastatic site for any sites with 4 or more metastases. We found that metastatic histology differed significantly based on metastatic site (Fisher-Freeman-Halton exact test, *p* < 0.001). Regional lymph node metastases were predominantly clear cell, whereas liver metastases demonstrated a higher proportion of sarcomatoid/rhabdoid histology and non-regional lymph nodes, adrenal gland, and lung metastases showed a mix (Figure 4).

**Figure 5:**
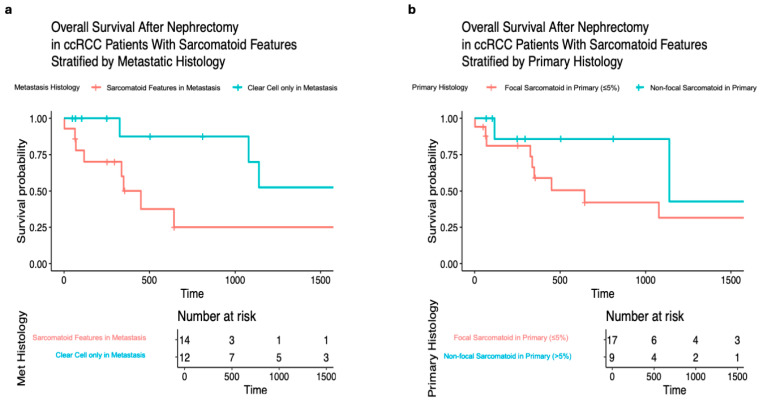
Kaplan–Meier curves showing overall survival based on (**a**) pure clear cell versus any degree of sarcomatoid/rhabdoid features in the metastasis and (**b**) focal versus any higher degree of sarcomatoid/rhabdoid features in the primary tumor.

Data regarding first-line systemic therapies were available for 15 patients (58%), with 11 patients receiving first-line immunotherapy-based regimens and 4 patients receiving first-line tyrosine kinase inhibitor (TKI) therapy. Ten of the fifteen patients in whom first-line systemic therapy data were available had documented progression of disease, with eight of the ten (80%) receiving subsequent-line systemic therapy. Kaplan–Meier curves were generated based on all patients to determine the impact of the primary histology and metastatic histology (Figure 5) on OS. Although not statistically significant, we noted a trend towards worse OS in patients with >5% sarcomatoid/rhabdoid in their primary compared to focal sarcomatoid, as well as in patients with any degree of sarcomatoid/rhabdoid in a metastatic lesion.

## 4. Discussion

In this study we examined a cohort of patients with ccRCC containing sarcomatoid or rhabdoid differentiation who underwent resection of metastatic disease. To our knowledge, this represents the largest cohort specifically examining metastatic histology in ccRCC with sarcomatoid differentiation. Our findings demonstrate that metastatic lesions arising from ccRCC/sRCC exhibit substantial histologic heterogeneity, ranging from pure clear cell morphology to pure sarcomatoid differentiation. These observations reinforce the concept that sarcomatoid differentiation represents a dynamic and heterogeneous process rather than a uniform or terminal phenotype.

We observed that increasing sarcomatoid/rhabdoid differentiation in the primary tumor was associated with a greater likelihood of sarcomatoid/rhabdoid morphology in metastatic lesions. ROC analysis demonstrated the proportion of sarcomatoid/rhabdoid differentiation in the primary tumor significantly predicted the presence of sarcomatoid/rhabdoid histology in metastases. While prior studies have suggested a threshold of approximately 30% sarcomatoid differentiation in the primary tumor as predictive of sarcomatoid metastases [6], our analysis identified an optimal cutoff of 10% in a cohort comprised exclusively of ccRCC/sRCC. This lower threshold found in our study may reflect biological differences between RCC subtypes, as previous studies included heterogeneous cohorts containing papillary, chromophobe, and unclassified RCC [6].

Despite this overall trend, our findings also highlight significant variability in metastatic histology. Several patients with predominantly sarcomatoid/rhabdoid primary tumors developed metastases composed entirely of clear cell carcinoma, while others with minimal sarcomatoid/rhabdoid differentiation in the primary tumor developed pure sarcomatoid/rhabdoid metastases. In addition, we observed occasional cases in which a single patient harbored multiple metastases with differing histologic composition, for example, one metastasis with pure clear cell histology and a second metastasis with sarcomatoid histology. These findings suggest that metastatic dissemination in ccRCC/sRCC is not strictly determined by the dominant histology of the primary tumor.

These observations raise important biological considerations. While sarcomatoid components were historically thought to possibly represent a distinct, more aggressive tumor clone, accumulating molecular and genomic evidence indicates that they instead reflect a dedifferentiated state arising from the underlying carcinoma. This process is thought to occur, at least in part, through an epithelial to mesenchymal transition, rather than representing a separate clonal lineage. Within this framework, our finding of substantial heterogeneity in metastatic histology becomes particularly informative. One possible explanation for this heterogeneity is that both epithelial (clear cell) populations and mesenchymal (sarcomatoid/rhabdoid) tumor cell populations may possess independent metastatic potential, allowing distinct phenotypic populations within the primary tumor to disseminate and establish metastases. Alternatively, these findings may reflect the increasingly recognized epithelial–mesenchymal plasticity in cancer biology, in which tumor cells may occupy hybrid EMT states that retain the capacity to disseminate while maintaining the ability to transdifferentiate into either epithelial or mesenchymal phenotypes after colonization of distant sites [7,8,9]. This concept of phenotypic plasticity provides a potential explanation for our observation that metastatic lesions can differ markedly from the dominant histology of the primary tumor, as well as from one another within the same patient. Although molecular studies are required to directly test these hypotheses, the histologic diversity observed in our cohort is consistent with a model of dynamic EMT plasticity.

We identified differences in metastatic histology based on metastatic site. Regional lymph nodes were significantly more likely to demonstrate clear cell morphology, whereas visceral metastases, particularly liver metastases, showed a greater proportion of sarcomatoid/rhabdoid differentiation. Mechanisms underlying this observation remain unclear; however, these findings raise the possibility that specific tumor phenotypes may preferentially seed or survive within particular metastatic environments. This could be due to differences in the local immune and stromal environment and is an important area of future research.

From a clinical perspective, the ability to predict metastatic histology may have therapeutic implications. Clear cell RCC and sarcomatoid RCC demonstrate distinct biological characteristics and responses to systemic therapy [2,10]. Clear cell tumors are frequently characterized by angiogenesis-driven signaling [11] and often respond to VEGF-targeted therapies [12], whereas sarcomatoid tumors exhibit greater immunogenicity [2] and may respond preferentially to immune checkpoint inhibition [4,13]. It is unclear whether primary tumor histology versus metastasis histology has greater implications for response to these treatments. Prior studies showing improved response of sRCC to immunotherapy have included tumors with variable degrees of sarcomatoid differentiation, and the histology of corresponding metastatic sites has not been well characterized. It is therefore possible that metastatic lesions with clear cell histology may respond more favorably to VEGF-targeted therapies, whereas those with sarcomatoid histology derive greater benefit from immunotherapy. In this context, our study suggests that a threshold of >10% sarcomatoid/rhabdoid features in the primary tumor may predict the presence of sarcomatoid histology in the metastatic sites. This finding could help stratify patients based on their likelihood of sarcomatoid differentiation in metastases and inform analyses of treatment response. As systemic treatment strategies continue to evolve, understanding the histologic composition of metastatic disease may become increasingly relevant for therapeutic decision-making.

Our study has several important limitations. First, the overall cohort size is relatively small, reflecting the rarity of sarcomatoid disease and the limited number of patients who undergo surgical resection of metastatic lesions. Consequently, only a small number of samples were available for each metastatic site, limiting our ability to assess site-specific influences on histology. Second, because some patients had multiple metastatic lesions, metastasis-level analyses, where each metastasis was analyzed individually, may introduce statistical dependence between observations. To mitigate this limitation, we also performed patient-level analyses that demonstrated similar overall trends. Additionally, our analysis combined tumors with both sarcomatoid and rhabdoid features. While sarcomatoid and rhabdoid histology are frequently grouped and described concurrently, we recognize that they may exhibit distinct implications on prognosis or therapeutic response. Future studies with larger cohorts are required to investigate the potential differences in sarcomatoid versus rhabdoid metastases. Finally, due to the limited sample size and heterogeneous treatment exposure, we were unable to perform multivariable analyses to fully evaluate clinical predictors of metastatic histology or survival. Despite these limitations, this study provides important insight into the histologic heterogeneity of metastatic disease in ccRCC/sRCC. By focusing on a cohort composed exclusively of ccRCC/sRCC, our findings help clarify patterns of metastatic histology within this biologically distinct group.

## 5. Conclusions

In summary, metastatic lesions arising from ccRCC with sarcomatoid/rhabdoid differentiation demonstrate substantial histologic heterogeneity, spanning the spectrum from pure clear cell carcinoma to pure sarcomatoid/rhabdoid morphology. The proportion of sarcomatoid/rhabdoid differentiation in the primary tumor is associated with the likelihood of sarcomatoid/rhabdoid features in associated metastatic lesions, although considerable inter- and intra-patient variability exists. Regional lymph node metastases were more likely to retain clear cell morphology, whereas visceral metastases demonstrated a higher prevalence of sarcomatoid/rhabdoid differentiation, suggesting potential site-specific influences on tumor phenotype.

These findings highlight the complexity of metastatic progression in ccRCC/sRCC and support a model in which metastatic histology is shaped by both intrinsic tumor biology and the surrounding microenvironment. Importantly, the observed heterogeneity may have clinical implications for prognostication and therapeutic decision-making, particularly in the era of immune checkpoint inhibitors, where tumor histology is increasingly relevant to treatment response. Taken together, these results underscore the need for further molecular studies to better define the mechanisms driving metastatic heterogeneity and to determine how these patterns may be leveraged to optimize patient stratification and therapy selection.

## Figures and Tables

**Table 1:** Patient demographics and metastatic characteristics.

Characteristic, *n* = 26	Value
Gender (*n*, %)	
Male	15 (57.7%)
Female	11(42.3%)
Age at time of nephrectomy (years)	62 (40–78)
BMI at time of nephrectomy	31 (22–44)
Metastatic site locations (% of total)	
Lymph node (regional)	29 (46.0%)
Lymph node (non-regional)	4 (6.3%)
Adrenal	8 (12.7%)
Liver	7 (11.1%)
Lung	4 (6.3%)
Bone	3 (4.8%)
Brain	1 (1.6%)
Chest wall	1 (1.6%)
Omentum	1 (1.6%)
Colon	1 (1.6%)
Duodenum	1 (1.6%)
Pituitary	1 (1.6%)
Scalp	1 (1.6%)
Psoas muscle	1 (1.6%)
Number of metastatic sites per patient	
One	3 (11%)
Two	15 (58%)
Three or more	8 (31%)

## Data Availability

De-identified data is included in the Supplemental Table S1. Further data is not shared due to ethical reasons to protect patient privacy.

## Supplementary Materials

The following supporting information can be downloaded at: https://www.mdpi.com/article/10.3390/jcm15103959/s1. Table S1. Primary and metastatic tumor characteristics per patient.
