# Supplementary material for: Histologic Heterogeneity of Metastases in Clear Cell Renal Cell Carcinoma with Sarcomatoid Differentiation
Source: J Clin Med. 2026 May 21;15(10):3959. doi: 10.3390/jcm15103959 (PMC13207079; doi:10.3390/jcm15103959)
Supplement: Supplementary file 1 [file jcm-15-03959-s001.zip › jcm-4241577-supplementary.pdf]

Table S1. Primary and metastatic tumor characteristics per patient.

Patient ID	% Sarcomatoid/rhabdoid in primary tumor	Presence of Rhabdoid features in primary tumor	Metastasis Location	Synchronous (S) or metachronous (M)	Metastasis Histology
1	<5	Present	Adrenal	S	Pure clear cell
2	<5	Present	Adrenal	S	Pure clear cell
			LN (regional)	S	Pure clear cell
			LN (regional)	S	Pure clear cell
			LN (regional)	S	Pure clear cell
			LN (regional)	S	Pure clear cell
			Pituitary	M	Pure clear cell
3	<5	Absent	LN (regional)	S	Pure clear cell
4	5	Absent	LN (regional)	S	Pure clear cell
			LN (regional)	S	Pure clear cell
			LN (regional)	S	Pure clear cell
			LN (regional)	S	Pure clear cell
			LN (regional)	S	Pure clear cell
5	5	Absent	Adrenal	S	Mixed, predominantly cc
			Liver	M	Pure clear cell
			Liver	M	Mixed, predominantly cc
6	5	Absent	Lung	S	Pure sarcomatoid
7	5	Absent	LN (regional)	S	Pure clear cell
8	5	Absent	LN (regional)	S	Pure clear cell
			Lung	S	Pure clear cell
9	5	Absent	LN (regional)	S	Pure clear cell
			LN (regional)	S	Pure clear cell
			Psoas Muscle	M	Pure clear cell
10	15	Present	Adrenal	S	Mixed, equal
			Liver	S	Mixed, equal
			Lung	S	Mixed, equal
11	15	Absent	LN (regional)	S	Mixed, predominantly cc
			LN (regional)	S	Mixed, predominantly cc
12	15	Present	LN (regional)	S	Mixed, equal
			Adrenal	S	Mixed, equal
13	15	Present	LN (non-regional)	S	Pure clear cell
14	40	Absent	Bone	M	Pure sarcomatoid
15	50	Present	Adrenal	S	Mixed, equal
			Lung	S	Mixed, equal
16	50	Absent	Bone	S	Pure clear cell
17	50	Present	Brain	M	Pure sarcomatoid
			Scalp	M	Pure clear cell

18	50	Absent	LN (non-regional)	S	Pure sarcomatoid
			Bone	M	Pure sarcomatoid
19	50	Absent	Chest wall	S	Pure clear cell
20	50	Present	LN (regional)	S	Pure sarcomatoid
21	80	Absent	Adrenal	S	Mixed, predominantly cc
			LN (regional)	S	Mixed, predominantly cc
			LN (regional)	S	Mixed, predominantly cc
			LN (regional)	S	Mixed, predominantly cc
			LN (regional)	S	Mixed, predominantly cc
			LN (regional)	S	Mixed, predominantly cc
			LN (regional)	S	Mixed, predominantly cc
			LN (regional)	S	Mixed, predominantly cc
			LN (regional)	S	Mixed, predominantly cc
			LN (regional)	S	Mixed, predominantly cc
22	80	Absent	LN (regional)	S	Pure clear cell
			LN (regional)	S	Pure clear cell
23	80	Absent	Adrenal	S	Pure sarcomatoid
			LN (non-regional)	S	Pure sarcomatoid
			Liver	S	Pure sarcomatoid
			Liver	S	Pure sarcomatoid
			Liver	S	Pure sarcomatoid
24	80	Present	Liver	S	Pure sarcomatoid
			Colon	S	Pure sarcomatoid
			Duodenum	S	Pure sarcomatoid
25	90	Absent	Omental	S	Pure sarcomatoid
26	90	Present	LN (non-regional)	M	Pure clear cell
